# The lncRNA *CASC15* regulates SOX4 expression in RUNX1-rearranged acute leukemia

**DOI:** 10.1186/s12943-017-0692-x

**Published:** 2017-07-19

**Authors:** Thilini R. Fernando, Jorge R. Contreras, Matteo Zampini, Norma I. Rodriguez-Malave, Michael O. Alberti, Jaime Anguiano, Tiffany M. Tran, Jayanth K. Palanichamy, Jasmine Gajeton, Nolan M. Ung, Cody J. Aros, Ella V. Waters, David Casero, Giuseppe Basso, Martina Pigazzi, Dinesh S. Rao

**Affiliations:** 1 Department of Pathology and Laboratory Medicine, UCLA, Los Angeles, USA; 2 Cellular and Molecular Pathology Ph.D. Program, UCLA, Los Angeles, USA; 3 Women and Child Health Department- Hematology-Oncology laboratory, University of Padova, Padova, Italy; 4 Molecular, Cellular and Integrative Physiology Ph.D. program, UCLA, Los Angeles, USA; 5 Microbiology, Immunology and Molecular Genetics Program, UCLA, Los Angeles, USA; 6 Medical Scientist Training Program, David Geffen School of Medicine, UCLA, Los Angeles, USA; 7 Jonsson Comprehensive Cancer Center, UCLA, Los Angeles, USA; 8 Broad Stem Cell Research Center, UCLA, 650 Charles E. Young Drive, Factor 12-272, Los Angeles, CA 90095 USA; 9 Present Address: Cedars-Sinai Medical Center, 8700 Beverly Blvd, Los Angeles, CA 90048 USA; 10 Present Address: Department of Pathology & Immunology, Washington University School of Medicine, St. Louis, MO 63110 USA; 11 Present Address: University of San Francisco, 2130 Fulton St, San Francisco, CA 94117 USA; 12 Present Address: All India Institute of Medical Sciences (AIIMS), New Delhi, India; 13 Present Address: Department of Molecular Cardiology Lerner Research Institute, 9500 Euclid Avenue. Cleveland, Cleveland, OH 44195 USA; 14 Present Address: Department of Molecular and Cell Biology, UC Berkeley, Berkeley, USA

**Keywords:** Non-coding RNA, CASC15, ETV6-RUNX1, SOX4, B-all

## Abstract

**Background:**

Long non-coding RNAs (lncRNAs) play a variety of cellular roles, including regulation of transcription and translation, leading to alterations in gene expression. Some lncRNAs modulate the expression of chromosomally adjacent genes. Here, we assess the roles of the lncRNA CASC15 in regulation of a chromosomally nearby gene, SOX4, and its function in RUNX1/AML translocated leukemia.

**Results:**

*CASC15* is a conserved lncRNA that was upregulated in pediatric B-acute lymphoblastic leukemia (B-ALL) with t (12; 21) as well as pediatric acute myeloid leukemia (AML) with t (8; 21), both of which are associated with relatively better prognosis. Enforced expression of *CASC15* led to a myeloid bias in development, and overall, decreased engraftment and colony formation. At the cellular level, *CASC15* regulated cellular survival, proliferation, and the expression of its chromosomally adjacent gene, *SOX4*. Differentially regulated genes following *CASC15* knockdown were enriched for predicted transcriptional targets of the Yin and Yang-1 (YY1) transcription factor. Interestingly, we found that *CASC15* enhances YY1-mediated regulation of the SOX4 promoter.

**Conclusions:**

Our findings represent the first characterization of this CASC15 in RUNX1-translocated leukemia, and point towards a mechanistic basis for its action.

**Electronic supplementary material:**

The online version of this article (doi:10.1186/s12943-017-0692-x) contains supplementary material, which is available to authorized users.

## Background

Among the several classes of non-coding RNA species being described, long non-coding RNAs are notable for their status as unique gene structures [1]. The majority of lncRNAs is characterized by capped, polyadenylated, and spliced transcripts that lack an open reading frame. Genes encoding lncRNAs show positional conservation in the genome and contain very short stretches of highly conserved sequences between species [1–3]. Despite the similarities in their genetic organization, there is a great deal of variation in the functions of different lncRNAs. They play a variety of roles at the cellular level, including regulation of transcription and translation, leading to alterations in gene expression. One of these functions is the regulation of gene expression in *cis*, which results in the modulation of expression of chromosomally adjacent genes upon knockdown or overexpression of lncRNAs [4].

Our recent work has identified a list of lncRNAs that are differentially expressed in pediatric B-lymphoblastic leukemia (B-ALL) patient samples [5]. One of the lncRNAs from our study, annotated as *CASC15*, (previously annotated as *LINC00340*) was of particular interest as it neighbors the protein coding gene, *SOX4*. SOX4 was first identified as a transcriptional activator in lymphocytes and plays an essential role in B-cell development [6, 7]. Recent studies have shown involvement of *SOX4* in many human malignancies, including the hematopoietic system [8, 9]. *CASC15* was recently described in two other types of cancer: neuroblastoma and melanoma. It is lost as part of the chromosome 6p22 deletion in neuroblastoma, and plays a role as a tumor suppressor gene in neuroblastoma cell lines [10]. Interestingly, a second study demonstrated that *CASC15* was associated with metastatic melanoma and siRNA-mediated knockdown resulted in altered growth and metastatic properties of melanoma-derived cell lines [11]. These two studies suggest somewhat different roles for *CASC15*, with an anti-proliferative phenotype in neuroblastoma, but a pro-metastatic role in melanoma.

In this study, we sought to delineate the function of *CASC15* in acute leukemia. *CASC15* expression was high in acute leukemia with *RUNX1* translocations, and its expression in cells led to increased apoptosis and decreased engraftment in the hematopoietic system. Our experiments also demonstrated the efficacy of knockdown of a lncRNA by using the recently described CRISPR/Cas9 system, adapted for this specific purpose. At the molecular level *CASC15* regulates SOX4 expression and downstream gene expression mediated by this transcription factor. Finally, the mechanism of action of CASC15 appears to involve modulation of gene expression by the transcription factor Yin and Yang-1 (YY1). These studies provide important insights into lncRNA function in the hematopoietic system.

## Methods

### Patients and samples

B- ALL patient samples were previously described [5]. Briefly, the B-ALL patient cohort consisted of children consecutively admitted to the Pediatric Oncologic Department at the University of Padua, Italy, from 2000 to 2008 with the diagnosis of B-ALL. A total of 125 patient samples used for qPCR consisted of 39 ETV6-RUNX1, 8 E2A-PBX, 16 MLL-rearranged, 3 BCR-ABL and 59 normal Karyotype B-ALL (the latter does not exclude sub-karyotypic rearrangement). The AML patient cohort consisted of 48 children consecutively admitted to the Pediatric Oncologic Department at the University of Padua (Padova, Italy) from 2000 to 2014 with the diagnosis of AML. Patients were molecularly diagnosed as previously described [12] and 8 carried the FLT3-ITD, 5 the Inv(16)CBFB-MYH11, 8 harbored the t(8;21)AML1-ETO, 12 MLL-rearrangements, and 12 were found negative for the molecular markers screened. All the procedures were approved by the local institutional review boards, and the study was considered exempt from review at UCLA.

### Cloning and cell culture

RS4;11 and MV4;11, (MLL-AF4-translocated; ATCC CRL-1873 and CRL-9591), REH (TEL-AML1-translocated; CRL-8286), 697 (E2A-PBX1-translocated), Nalm-6, and 70Z/3 (ATCC TIB-158) murine pre-B cell leukemic cell line, and the HEK 293 T cell line (ATCC CRL-11268) were grown as previously described [5, 13]. mmu-miR-155 formatted siRNAs were cloned into BamHI and ApaI or XhoI in the pHAGE2-CMV-ZsGreen-WPRE vector [5]. To determine the 5′ and 3′ transcript ends of the lincRNAs, we performed RACE (Rapid Amplification of cDNA Ends) using FirstChoice RLM-RACE kit (Ambion). Using the sequence information from 5′ and 3′ RACE products, we cloned full length transcripts into an MSCV viral vector, as described previously. Primer sequences used in cloning are listed in Additional file 1: Table S1. For CRISPR-Cas9 mediated targeting, guide RNAs were designed using the Zhang lab website (http://crispr.mit.edu/) [14]. Guide RNA sequences are listed in the Additional file 1: Table S1. Lentiviral production was done in HEK293T cells using the helper plasmids pMD2G and psPAX2. REH cells were spin-infected at 30 °C for 90 min in the presence of polybrene. Cells were selected with 5 μg/mL of puromycin for 7 days. Cell culture was performed as previously described [15]. Flow cytometry was used to detect apoptosis using Annexin V staining [5].

The paired gRNA MSCV retroviral vector (Manuscript in preparation) was constructed via standard cloning techniques to contain two U6-gRNA cassettes in tandem followed by a EFs-mCherry reporter cassette, with each of the three cassettes separated by a short spacer sequence. The hU6 promoter, gRNA scaffold, and EFs promoter elements were derived from the pLentiCRISPRv2 vector [16]. The mU6 promoter was designed from the GenBank sequence NC_000076.6 (nt 79,908,880–79,909,195). A silent mutation was incorporated into the mCherry reporter element [17] to remove an internal *Bbs*I restriction site. The 20-nt gRNA sequences flanking a 3.1–3.8 kb region (encompassing the first two exons) of mouse *Casc15* (*2610307P16Rik*) were designed using sgRNA Scorer with CasFinder [18]. Detailed methods are available upon request.

### Transduction and cell sorting

Lentivirus and MSCV-based retroviral vectors were produced to generate knockdown and overexpression constructs as previously described [15, 19]. 5.0 × 10^5 cells were spin-infected twice at 30 °C for 90 min in the presence polybrene (4 μg/mL). Transduced cell lines were sorted for GFP positivity using a BD FACSAriaI/II cell sorter.

### RT-qPCR

RT-qPCR was performed as previously described [19, 20]. For AML patient samples RT-qPCR experiments were carried out using SYBR Green PCR Master Mix (Applied Biosystems) with an ABI 7900 Real-Time PCR System (Applied Biosystems). Beta-glucuronidase (GUS) was used as endogenous control. PCR reaction was performed in three replicates, the comparative threshold cycle (dCt) method [21] was used to analyze results. For all other assays, the results were expressed as fold-change with normalization to GAPDH or Actin. Primer sequences used are listed in Additional file 1: Table S1. Primer set #1 for CASC15 was used for all assays except for those with enforced overexpression of the long isoform.

### Microarray data analysis

RNA samples from RS4;11 control and siRNA-knockdown cells were DNAse-treated and column purified using RNeasy MiniElute Cleanup Kit. Samples were hybridized at the UCLA microarray core facility using Affymetrix HG-U133_Plus_2 microarray. The Affymetrix raw data files (.cel files) were loaded into the R program for quality control analysis. Additionally, raw hybridization intensities were normalized using the MAS5 method the Affy package in R. Normalized values were sorted by detection *p*-value ≤0.05. Differential expression analysis was performed using unpaired Bayesian comparison model (CyberT Website) [22]. Genes with a PPDE ≥99% and a fold change ≥2 was used for the further analysis. For the analysis of REH control and knockout cells, we utilized a slightly different analysis. For differential analysis the raw data files were uploaded into the R environment and analyzed using the R library of Linear Models for Microarray Data (LIMMA). Pairwise comparison and eBayes fit was carried out [13] Analysis of differentially expressed genes was carried out using WebGestalt and GSEA [23, 24]. All data files are available in the NCBI Gene expression Omnibus database under accession number GSE101149.

Microarray data deposited in NCBI’s Gene Expression Omnibus database (GEO; http://www.ncbi.nlm.nih.gov/geo/) through the GEO accession number GSE17459 [25], 33 ETV6-RUNX1, 7 BCR-ABL, 31 DS-ALL, and 31 hyperdiploid ALL patients) and GSE75461 [26] (16 RUNX1-RUNX1T1, 8 FLT3-ITD, 4 CBFB-MYH11, 7 MLL-rearranged, 24 Negative, 7 DEK-NUP214, and 19 NUP98-translocated patients) were used to validate CASC15 expression levels in these two independent cohorts of B-ALL and AML pediatric patients, respectively.

### Bone marrow transplant

As previously described, 5-FU enriched bone marrow was spin-infected twice with MGP vector or M-Casc15-G [27]. Lethally irradiated recipient mice were injected with transduced donor bone marrow at least 6 h after irradiation. 8 mice were used per group. These mice were bled every 4 weeks post transplantation. All mice were housed under pathogen free conditions at the University of California, Los Angeles.

### Flow cytometry

Bone marrow, blood and spleen were collected from the mice at 16–27 weeks post-transplantation. Cells were lysed in RBC (red blood cell) lysis buffer. Fluorochrome conjugated antibodies were used for staining (Biolegend). After 30–40 min of staining at 4 °C, cells were washed twice with PBS and fixed with 1% PFA in FACS buffer. Flow cytometry was performed at the UCLA Jonsson Comprehensive Cancer Center (JCCC) and at the BROAD Stem Cell Research Flow Core. Analysis was performed using FlowJo software. A list of antibody combinations used to define hematopoietic subsets is provided in Additional file 2: Table S2.

### CFU assay

5-Fluorouracil treated mice were sacrificed after 5 days and the bone marrow was plated in media supplemented with IL-3, IL-6 and mSCF (MethoCult 03434). After 24 h of plating, the bone marrow was infected twice with retroviruses expressing the empty MGP vector or CASC15/Casc15. GFP + cells were sorted and plated at low concentration on methylcellulose medium containing mSCF, mIL3, hIL-6 and hEpo.

#### Luciferase assay

Approximately 2000 bp upstream from the transcription start site of SOX4 was cloned upstream of firefly luciferase reporter gene in the pGL4.11 vector. pGL4.75 Renilla luciferase vector was used for normalization. HEK 293 T cells were transfected with the pGL4.75 and pGL4.11 containing reporter vectors at a 1:10 ratio (10 ng:100 ng), along with a combination of pCMV (empty or YY1: Origene-SC118004) and MSCV (empty or CASC15) vectors at a 1:5 ratio (10 ng:50 ng), or a modification thereof. BioT (Bioland Scientific LLC) was used in 24-well plates as per the manufacturer’s instructions. Cells were lysed after 36–48 h and supernatant lysate was collected as per manufacturer’s instructions (Promega). The dual luciferase assay kit (Promega) was used as substrates for Renilla and firefly luciferase activity. The ratio of firefly to Renilla luciferase activity was calculated for all samples and normalized to the vector control.

#### Cell fractionation

Cells were pelleted and resuspended in NP-40 lysis buffer (0.5% NP-40, 10 mM Tris (pH 7.4), 10 mM NaCl, 3 mM MgCl2, and 1 mM DTT), following which they were incubated on ice for 5–10 min [5]. Suspension was spun at 1200 rpm at 4 °C for 5 min. Supernatants consisting of the cytoplasmic fraction were transferred to a new tube without disturbing the nuclear pellet. Each fraction was resuspended in Trizol and RNA extractions were carried out.

#### RNA immunoprecipitation (RIP)

HEK 293 T cells were grown in a 10 cm plate and transfected with 7.5 μg of MSCV control or CASC15 plasmid and 7.5 μg of pCMV control or YY1 plasmids with Bio-T transfection reagents. RIP was done as previously described [3]. The procedure was carried out under RNAse-free conditions. 10 million cells were harvested and incubated for 20 min on ice in 2 ml of nuclear isolation buffer (1.28 M sucrose, 40 mM Tris HCL pH 7.5; 20 nM MgCl2, 4% triton X100), 2 ml of PBS and 6 ml of water. Nuclei were pelleted and resuspended in 1 ml of RIPA (protease inhibitor added). The lysate was homogenized for 15–20 strokes with the Dounce homogenizer and centrifuged for 13,000 rpm for 10 min to pellet the debris. Input fraction was removed for RNA and protein isolation. The rest of the supernatant was pre-cleared with normal IgG (1 μg) and protein A/G plus beads (Santa Cruz) for 1 h at 4 °C. Beads were pelleted by centrifugation 2500 rpm for 5 min 4 °C. Pre-cleared lysate was divided into two and incubated overnight with gentle rotation at 4 °C with the YY1 antibody (Rabbit mAb #2185: Cell signaling) and the normal IgG. A/G plus agarose beads were added and incubated for another 1–2 h. Beads were pelleted and washed 3 times with RIPA buffer and once with PBS. Supernatant was divided into 3:1 ratio for RNA and protein extraction. Mouse monoclonal anti-YY1 was used for the western blot (Abgent: AM2231b) on the immunoprecipitate.

## Results

### *CASC15* is overexpressed in acute leukemia with RUNX1 translocations and encodes multiple splice variants

**Fig. 1:**
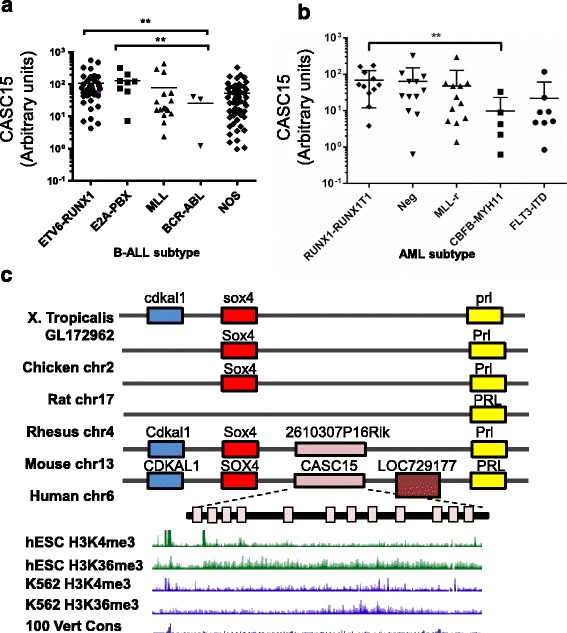
*CASC15* expression is highest in acute leukemia samples that carried a translocation involving the RUNX1/AML1. **a** RT-qPCR of the B-ALL patient bone marrow samples using primer set #1 normalized to Actin, showing differential expression between cytogenetic subtypes based on translocations (*n* = 125). Comparisons were made using 1-way ANOVA (*p* < 0.02) and a two-tailed T-test; statistically significant differences are denoted as follows: *P* < 0.005 (**)). **b** RT-qPCR analysis of *CASC15* expression in different molecular and cytogenetic subtypes of AML patient samples, using primer set #1 and normalized to GUS (*n* = 48). Comparisons were made using a two-tailed T-test. **c** *Top*: Schematic showing the genomic conservation of the syntenic block among vertebrates. Highly conserved region of the human *CASC15* was used for the analysis. *Bottom*: Chip-seq histone modification map from the ENCODE/Broad institute, taken from UCSC genome browser, shows H3K4me3 and H3K36me3 patterns at the *SOX4* and *CASC15* locus in two different cell types indicating active transcription of the lncRNA

In our previous study, we identified *CASC15* as a differentially expressed lncRNA between three subtypes of pediatric B-ALL [5]. *CASC15* showed a significant variation in expression level depending on the subtype of B-ALL in a larger set of B-ALL cases by RT-qPCR (Fig. 1a, 1-way ANOVA, *p* < 0.02). Both ETV6-RUNX1 and E2A-PBX subtypes had significantly higher *CASC15* expression compared to the BCR-ABL subtype (Fig. 1a). We also quantified *CASC15* expression in 48 bone marrow aspirates from pediatric acute myeloid leukemia (AML) that showed 4 different translocations (Fig. 1b). Overall expression of *CASC15* showed a trend toward higher expression in samples with t(8;21) translocation. However, statistical significance is reached only when compared to the samples with inv.(16) (Fig. 1b). To validate higher *CASC15* expression in t(8;21) and t(12;21) rearranged leukemia, we analyzed two datasets deposited in NCBI’s Gene Expression Omnibus database (GEO) (*N* = 102 ALL, *N* = 85 AML; GSE17459 and GSE75461) [25, 26]. *CASC15* was upregulated in *ETV6-RUNX1*-translocated patients compared to those with Down Syndrome ALL (DS-ALL) and hyperdiploid ALL (Additional file 3: Figure S1a; 1-way ANOVA, *p* < 0.01). In AML patients, we confirmed *CASC15* upregulation in *RUNX1-RUNX1T1*-translocated patients compared with those with inv(16) and *DEK-NUP214* translocations (Additional file 3: Figure S1b). It is interesting to note that the expression of *CASC15* is highest in AML cases with t(8;21), and ALL cases with t(12;21), which have the common translocation partner *AML1/RUNX1*. *CASC15* expression was not statistically associated with survival by Kaplan-Meier analysis or in a multivariate regression model (Additional file 3: Figure S1f).

To fully characterize the transcript structure of *CASC15*, we carried out 5’RACE and 3’RACE (Rapid Amplification of cDNA Ends) (Additional file 3: Figure S1c-e). Using the sequence information from the 5′ and 3′ RACE products, we cloned short (S) and a long (L) isoforms of the full length *CASC15* transcripts. The *CASC15* (L) transcript is ~1634 nucleotides in length derived from 11 exons, while the *CASC15* (S) transcript is ~1193 nucleotides in length derived from 7 exons (Additional file 3: Figure S1g and Additional file 1: Table S1). ChIP-Seq data at the *CASC15* locus obtained from the Encode project showed that the chromatin signatures consisted of H3K4me3, usually present in promoter regions, and H3K36me3, corresponding to transcribed gene bodies indicating that it is a transcriptional element (Fig. 1c) [1]. Inspection of the annotated mouse transcriptome revealed the presence of a long non-coding RNA (RIKEN cDNA 2610307P16) sharing 64% similarity with human *CASC15*, with 86% sequence identity at the 5′ end (Fig. 1c). Subcellular fractionation experiments revealed that *CASC15* is predominantly a nuclear-localized lncRNA in all B-ALL cell lines tested (Additional file 3: Figure S1i-k). In this study, we utilized NALM6, REH, and RS4;11 cells, which show low (NALM6) and high (REH and RS4;11) levels of *CASC15* expression, to analyze gain-of-function and loss-of-function phenotypes, respectively.

### *CASC15* overexpression opposes cellular proliferation and promotes myeloid bias in vivo

**Fig. 2:**
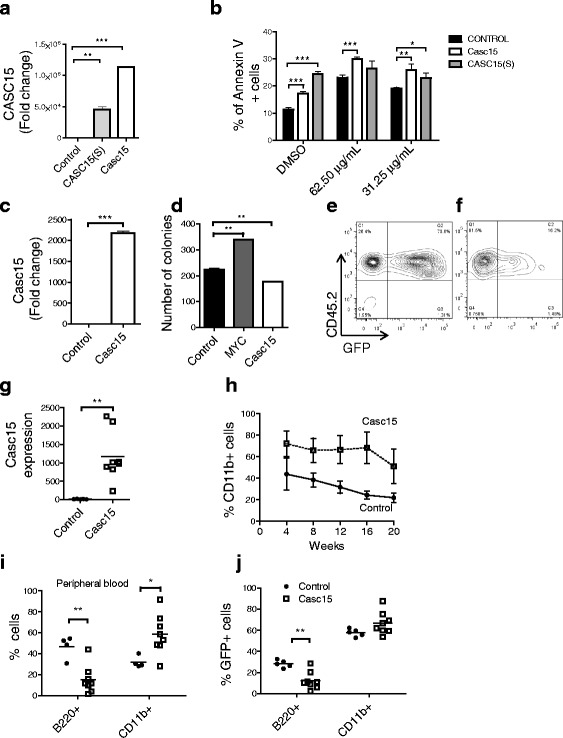
Over-expression of *CASC15* leads to increased apoptosis in B-ALL cells and myeloid expansion in mice. **a** RT-qPCR of mouse pre-B 70Z/3 cell line transduced with either human *CASC15(s)* or murine *Casc15*. **b** Annexin V staining of 70Z/3 stably transduced with *CASC15*(S) and mouse *CASC15*, either at baseline (DMSO) or treated with prednisolone (concentrations as indicated). **c** RT-qPCR or primary murine bone marrow cells transduced with murine *Casc15*. **d** Methylcellulose colony formation assay using MYC (positive control) or murine *Casc15*. **e**-**f** FACS analysis of peripheral bleed from mice 4 weeks after bone marrow transfer showing successful engraftment and transduction (GFP+) in vector (**e**) and *CASC15* (**f**) over expression mice. **g** RT-qPCR showing *Casc15* overexpression in bone marrow at 16 weeks after transplantation. **h** FACS analysis of peripheral bleeds from the mice 4–20 weeks after bone marrow transplantation showing increased percentage of myeloid cells in the *Casc15* overexpression mice. **i** FACS analysis of peripheral blood showing percentage of B220+ and CD11b + cells at 16 weeks post-transplantation. **j** FACS analysis of bone marrow showing percentage of GFP+, B220+ and GFP+, CD11b + cells at 16 weeks post-transplantation. In experiments depicted for **g**-**j**, *n* = 8 mice/group, and experiments were repeated three times. Statistically significant differences are shown as follows: *p* < 0.05 (*); *p* < 0.01 (**); *p* ≤ 0.0005 (***). All qPCR analyses for CASC15 were performed with primer set #1 and normalized to GAPDH or actin, except where otherwise noted. Experiments were repeated three times for validation

To examine the effects of *CASC15* gain of function, murine *Casc15* (Additional file 3: Figure S1h) was cloned into a MSCV based dual promoter vector. Indicating a conserved function, enforced expression of either the murine or human lncRNA in mouse pre-B cell line 70Z/3 resulted in increased apoptosis at basal levels and after prednisolone treatment (Fig. 2a, b). In human NALM6 cells, the effects were less pronounced (data not shown). In contrast to overexpression, knockdown of *CASC15* resulted in decreased levels of prednisolone-induced apoptosis in RS4;11 cells (Data not shown).

To examine the role of *CASC15* in the hematopoietic system, we undertook gain-of-function studies in primary bone marrow cells. Enforced expression of *Casc15* in 5-FU enriched bone marrow resulted in decreased overall colony formation in a methylcellulose assay (Fig. 2c, d). Next, we transplanted 5-FU enriched bone marrow transduced with retrovirus expressing *Casc15* into lethally irradiated recipient mice. FACS analysis of peripheral blood from mice with *Casc15* overexpression showed overall decreased engraftment (Fig. 2e, f) and increased myeloid cells as a percentage of the GFP+ population (Fig. 2h). This phenotype was persistent and the relative myeloid bias was maintained in *Casc15* expressing cells throughout the course of the experiment. At the end of the experiment, hematolymphoid organs were analyzed, revealing overall reduced engraftment, and a myeloid bias to development (Fig. 2i-j; Additional file 4: Figure S2c, S2i). Given the relative decrease in bone marrow B-cells, we analyzed the developmental pathway of B-cells in the bone marrow [28]. Among the Hardy fractions, we observed an overall decrease in the frequency of cells in all fractions with a significant decrease in fractions B, D and E/F, but no changes in earlier precursors (Additional file 4: Figure S2e-g). These findings indicate that enforced expression of *Casc15* causes an overall reduction in hematopoiesis, with the highest reduction in B-cell development. Taken together, our findings imply that *Casc15* is pro-apoptotic and reduces hematopoietic engraftment, particularly B-cell development, in vivo.

### *CASC15* regulates the expression of *SOX4*

**Fig. 3:**
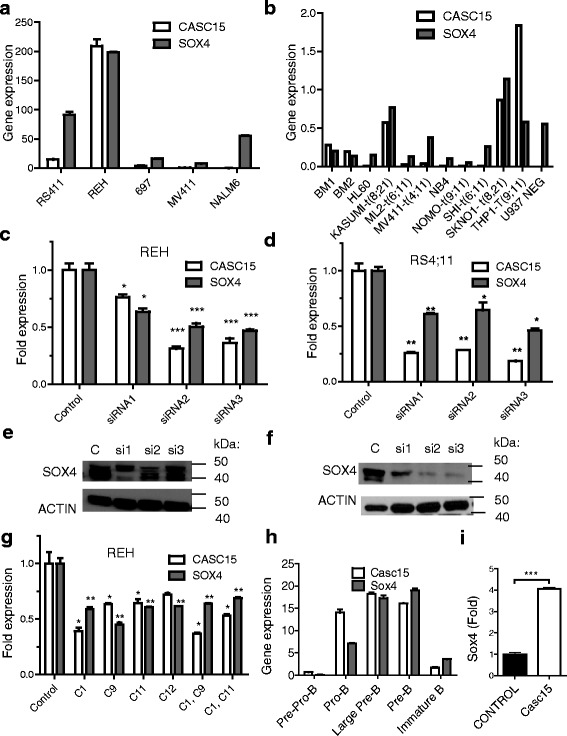
*CASC15* expression is strongly correlated with and regulates *SOX4* expression. **a**-**b** Correlation between *CASC15* and *SOX4* expression in various B-ALL and AML cell lines. **c**-**d** RT-qPCR of *CASC15* and *SOX4* in REH cells (**c**) and RS4;11 (**d**) following knockdown of *CASC15* using three independent siRNA sequences. Statistically significant differences from control are noted as follows: *p* < 0.05 (*); *p* < 0.01 (**); *p* < 0.0005 (***). **e**-**f** Western blot analysis of SOX4 protein levels in RS4;11 (**e**) and REH (**f**) cell lines upon *CASC15* knockdown. (**g**) RT-qPCR of *CASC15* and *SOX4* in REH cells following CRISPR/Cas9 mediated targeting. Statistically significant differences from control are denoted as follows: *p* < 0.05 (*); *p* < 0.01 (**). **h** RT-qPCR of murine *Casc15* and *Sox4* on RNA extracted from FACS-purified hematopoietic progenitor fractions shows a rough correlation in their expression, particularly in the B-cell subsets. **i** RT-qPCR for murine *Sox4* following transduction of bone marrow cells with murine *Casc15* shows upregulation of expression. All qPCR analyses for CASC15 were performed with primer set #1 and normalized to GAPDH or actin, except where otherwise noted. Experiments were repeated three times for validation. Experiments were repeated three times for validation

Some lncRNAs regulate the expression of chromosomally neighboring genes - a phenomenon typically observed when positional conservation is present in vertebrate species. The immediately adjacent gene to *CASC15*, *SOX4*, is a transcription factor that is involved in B- cell development as well as B- cell malignancies. In B-ALL patient samples, *SOX4* and *CASC15* had a positive correlation in B-ALL with TEL-AML1 translocations with an R^2^ value of 0.324 (Additional file 5: Figure S3b). We also observed that *CASC15* and *SOX4* expression showed a correlation in their expression levels in both B-ALL cell lines (Fig. 3a) and AML cell lines (Fig. 3b). *CASC15* expression was noted to be highest in REH, KASUMI, SKNO1, that carry RUNX1 translocations, consistent with the primary patient data, as well as in THP1 cells that carry a translocation of MLL. Confirming the idea of a positive correlation, RNA-sequencing data from the Cancer Cell line Encyclopedia (CCLE) [29] showed that B-ALL and AML cell lines showed a strong positive correlation between the expression of CASC15 and SOX4, while DLBCL and non-hematopoietic tumor cell lines did not (Additional file 5: Figure S3c). To understand whether a causative relationship existed between the expression of *CASC15* and *SOX4*, we designed and validated siRNAs to knockdown *CASC15* in REH and RS4;11 cells. We noted that knockdown of *CASC15* also resulted in a knockdown of *SOX4* (Fig. 3c-f). To further characterize this relationship, we edited *CASC15* by CRISPR/Cas9 [30, 31]. We targeted the transcription start site, intron-exon boundaries and the polyadenylation signal with a series of guide RNAs (C1, C9, C11, and C12) (Additional file 5: Figure S3e and Additional file 1: Table S1). Heteroduplex DNA that resulted from Cas9:sgRNA-mediated cleavage was observed by the endonuclease T7 assay (Additional file 5: Figure S3g-j). RT-qPCR data showed that targeting of specific splice junctions resulted in down regulation of *CASC15* in REH cells (Fig. 3g) and RS4;11 cells (Additional file 5: Figure S3f). Concurrent with downregulation of *CASC15*, CRISPR/Cas9 mediated knockout also lead to downregulation of *SOX4* (Fig. 3g). To assess species conservation, we sorted mouse bone marrow into progenitor subsets and examined *Casc15* and *Sox4* by RT-qPCR [28, 32]. *Casc15* is expressed in all the hematopoietic compartments tested, however, the highest expression was observed at the common lymphoid progenitor (CLP) stage and pro-B to pre-B stages (data not shown, Fig. 3h and Additional file 6: Figure S4). *Sox4* shows a similar expression profile in committed B-cell progenitors (Fig. 3h). Complementing this data, bone marrow transduced with a retrovirus overexpressing *Casc15* demonstrated increased *Sox4* levels (Figs. 2c and 3i). Together these data strongly suggest that *CASC15* regulates *SOX4* levels, particularly in the loss-of-function context.

**Fig. 4:**
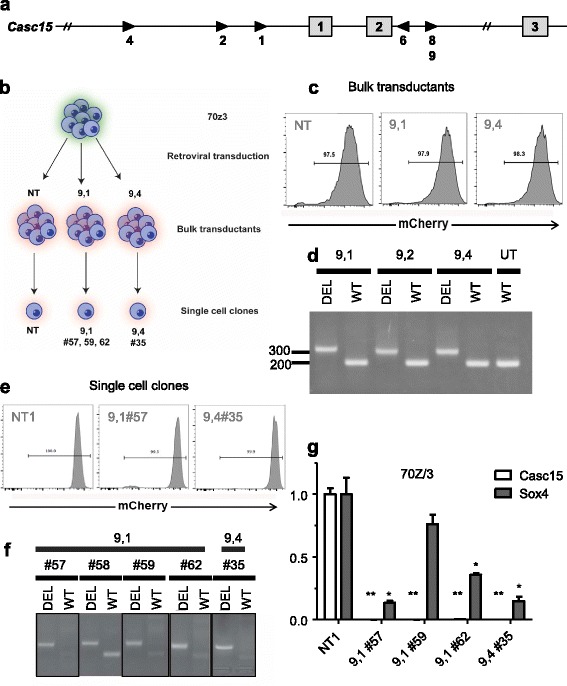
*Casc15* knockout in murine cells results in *Sox4* downregulation. **a** Schematic of *Casc15* gene in the mouse showing exonic sequences 1 and 2, along with the placement of short guide RNAs to induce deletion of these exons. **b** Schematic of approach to generation of genomic-level knockouts of *Casc15*. **c** FACS analysis of mCherry expression ion bulk transductants from this approach. **d** PCR analysis of mutant (DEL) and wild-type (WT) *Casc15* alleles in bulk transductants cells. **e** FACS analysis of mCherry expression in single cell clones generated by limiting dilution plating. **f** PCR analysis of mutant (DEL) and wild-type (WT) *Casc15* alleles in single cell clones generated by limiting dilution plating. **g** RT-qPCR analysis of *Casc15* and *Sox4* expression. Statistically significant differences from control are noted as follows: *p* < 0.05 (*); *p* < 0.01 (**)

To further confirm our findings, we designed a second CRISPR/Cas9-based method to knockout *Casc15*. For this purpose, we designed and built a MSCV-based retrovirus that contains a mCherry reporter and two U6-driven cassettes for the expression of small guide and scaffold RNA. To knockout *Casc15*, we targeted exons 1 and 2 with a series of small guide RNAs. Three guide RNAs (1,2, and 4 on the 5′ end; 6, 8, 9 on the 3′ end) were designed on each side of the knockout region (Fig. 4a). Next, we retrovirally transduced murine 70Z/3 cells with constructs containing pairs of guide RNAs, resulting in bulk transductants, that were subsequently subjected to single cell cloning (Fig. 4b). Measurement of mCherry fluorescence by FACS in the bulk transductants confirmed efficient transduction (Fig. 4c). To genotype the bulk transductants, we utilized two sets of primers, one to detect the deleted allele (DEL) and the other to detect the wild-type allele (WT). As anticipated, the initial bulk transductants showed the presence of both alleles of *Casc15,* reflecting a mixture of cells with wild-type, heterozygous, and homozygous deletion of *Casc15* (Fig. 4d). Then, we subjected the bulk transductants to single cell cloning by limiting dilution plating. As can be seen, the single cell clones retained strong mCherry fluorescence (Fig. 4e), and we identified several clones that contained a homozygous deletion of Casc15, including the clones shown (Fig. 4f). As expected, RT-qPCR showed a complete absence of Casc15 and all the clones showed reduced expression of Sox4, which was statistically significant except in one case. Hence, we have confirmed a causal relationship between Casc15 and Sox4 using three different knockdown/knockout systems.

### *CASC15* knockdown leads to enrichment for the transcriptional targets of YY1

**Fig. 5:**
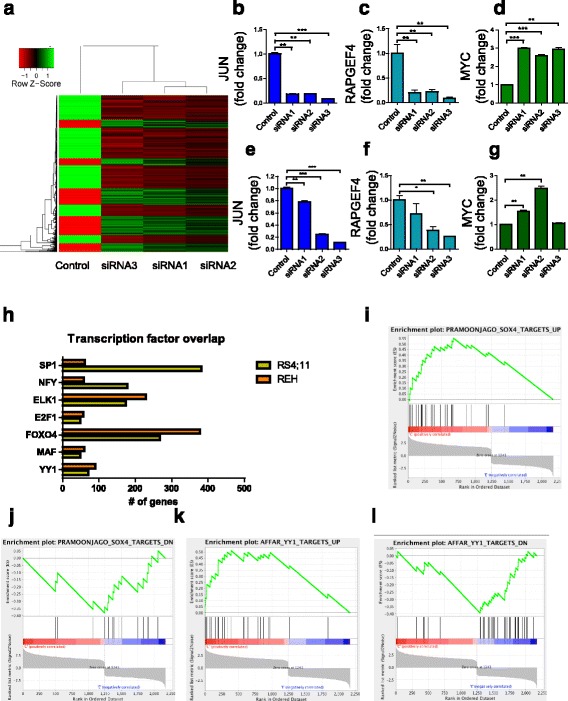
*CASC15* knockdown leads to enrichment for the transcriptional program of *SOX4* and *YY1*. **a** Unsupervised hierarchical gene clustering of differentially expressed genes upon *CASC15* siRNA mediated knockdown in RS4;11 cells (PPDE >99%, fold change >2). **b**-**g** RT-qPCR confirmation of differentially expressed genes from the microarray in RS4;11 (**b**-**d**) and REH (**e**-**g**) cell lines. **h** To narrow down transcription factors that might be responsible for the observed changes in gene expression, we overlapped transcription factors that were associated with the differentially expressed gene sets in *CASC15* KD RS4;11 cells as well as *CASC15* KO REH cells. Shown are the numbers of genes in the differentially expressed gene set for each transcription factor that showed an association. **i**-**j** Enrichment plots from gene set enrichment analysis (GSEA). The differentially regulated gene set from *CASC15* KD RS4;11 cells showed a positive enrichment score when compared to genes up-regulated in ACC3 cells with *SOX4* knockdown (**i**; Enrichment Score = 0.5, FDRq = 0.0 and *P* value = 0.0), and showed a negative enrichment score when compared to genes downregulated in ACC3 cells with SOX4 knockdown (**j**; Enrichment Score = −0.38,FDRq = .017 and *P* value = 0.01). **k**-**l** Enrichment plots from gene set enrichment analysis (GSEA) showing that the differentially regulated gene set showed a positive enrichment score when compared with upregulated genes upon YY1 knockdown (**k**; Enrichment Score = 0.5, FDRq = 0 and *P* value = 0.0) and a negative enrichment score with downregulated genes upon YY1 knockdown (**l**; Enrichment Score = -0.39, FDRq = 0 and *P* value = 0.0)

To gain insight into the molecular mechanism by which *CASC15* functions, we examined gene expression in RS4;11 cell lines where *Casc15* was knocked down (Fig. 5a). Upon siRNA mediated knockdown (*CASC15* KD) of *CASC15*, 3289 microarray probes showed differential expression with PPDE >99%, fold change >2. In agreement with the RT-qPCR, *SOX4* was downregulated in *CASC15* KD cell lines, and gene set enrichment analysis [33] demonstrated that genes that are known to be downstream of *SOX4* [34] were also regulated by *CASC15* knockdown, confirming a downstream effect on *SOX4* transcriptional activity (Fig. 5i-j). To validate the microarray, RT-qPCR was used to confirm the expression pattern of three genes that were highly differentially regulated in RS4;11 (Fig. 5b-d) and REH (Fig. 5e-g) *CASC15* KD cell lines. Functional enrichment of differentially expressed genes was carried out using WebGestalt. In parallel, we also carried out microarray analysis of REH cells with CRISPR/Cas9-mediated deletion of *CASC15* (*CASC15* KO; Additional file 7: Figure S5). Significant changes were seen in several categories of genes in both datasets when analyzed by WebGestalt [35] (Additional file 7: Figure S5a). Because of *CASC15*’s nuclear localization, we hypothesized that the downstream effects may be mediated by a transcription factors. This analysis led us to a list of transcription factors that were putatively responsible for the observed changes in gene expression in both REH and RS4;11 cell lines (Fig. 5h). Amongst these genes, the *YY1* transcription factor showed consistent changes between cell lines, was statistically enriched, and is known to bind to the *SOX4* promoter [36]. GSEA analysis confirmed that both SOX4 and YY1 transcriptional targets [34, 37] are also enriched in our differentially expressed gene set (Fig. 5k-i and Additional file 7: Figure S5c), reinforcing the idea that *CASC15* may act through transcriptional regulation of a specific transcription factor, such as YY1, with a downstream effect on SOX4.

### YY1 mediated transcriptional regulation of SOX4 promoter is promoted by CASC15

**Fig. 6:**
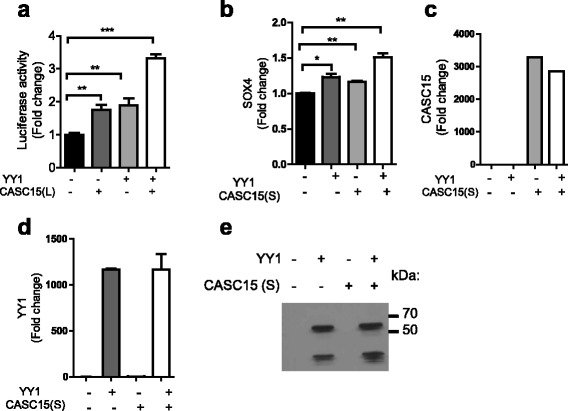
*CASC15* regulates the activity of YY1 on the *SOX4* promoter. **a** Transcriptional activity of *SOX4* promoter upon *CASC15* (L) and/or *YY1* overexpression, as measured by dual luciferase assay. Luciferase values are normalized to the empty vector. **b**-**d** RT-qPCR with primers directed against SOX 4 (b) *CASC15* (**c**) and *YY1* (**d**) and Western blot for YY1 (**e**) in 293 T cells transiently transfected with *YY1* and *CASC15*. All qPCR analyses for CASC15 were performed with primer set #1 and normalized to GAPDH or actin, except where otherwise noted. Experiments were repeated three times for validation

To further elucidate the relationship of *CASC15* and YY1 to regulation of SOX4 promoter, we cloned approximately 2000 bp upstream region of the SOX4 promoter into the luciferase reporter vector, pGL4.11 [38]. This region contains three putative binding sites for the transcription factor YY1 [36]. Dual luciferase reporter assays in HEK 293 T cells, with constitutive expression of *CASC15*, caused increased SOX4 promoter activity (Additional file 7: Figure S5d). Notably, when *CASC15* and YY1 were co-expressed, SOX4 promoter activity was further enhanced (Fig. 6a). Transient transfection of 293 T cells consistently led to expression of YY1 mRNA, CASC15 RNA, and YY1 protein (Fig. 6c-e). In addition, in these transient transfection assays, *SOX4* mRNA was upregulated, indicating an effect on the endogenous *SOX4* locus (Fig. 6b). These data show a direct functional impact of CASC15 and YY1 on transcriptional regulation of SOX4.

Recent work has indicated that one mechanism of lncRNA-mediated gene regulation is via interaction with transcriptional and/or epigenetic regulators, such as the Polycomb repressor complex or indeed YY1 itself [39, 40]. Furthermore, given that YY1 has a role in regulating PRC and can tether non-coding RNA to chromatin [41, 42], we performed an RNA immunoprecipitation (RIP) assay using anti-YY1 antibodies in 293 T cells transiently transfected with combinations of *YY1* and *CASC15* (Additional file 7: Figure S5e). Following successful transfection and immunoprecipitation of YY1, we found that *CASC15* was modestly enriched in RIP specifically with the anti-YY1 antibody (Additional file 7: Figure S5e). However, RIP assays performed against endogenous YY1 in REH cells were unsuccessful, likely due to a low level of endogenous YY1 under baseline growth conditions (data not shown). Nonetheless, our data shows that YY1 enhances transcription from the *SOX4* promoter, and that *CASC15* appears to enhance transcriptional upregulation of *SOX4* mRNA.

## Discussion

The field of lncRNA research has recently been growing and the diversity of functions ascribed to lncRNAs includes transcriptional regulation amongst several others [1, 3, 43]. Following up on our initial discovery of dysregulated lncRNA expression in B-ALL, we have now characterized individual lncRNAs. Interestingly, the expression of *CASC15* in B-ALL and AML samples was highest in cases that carried a translocation involving the *RUNX1/AML1*. *RUNX1* mutations and translocations are amongst the most commonly seen mutations in hematologic malignancies [44–46]. All of these alterations lead to loss-of-function of *RUNX1* by a variety of mechanisms. It will be of interest to examine *CASC15* in other leukemia subtypes with *RUNX1* loss-of-function, and these experiments are the focus of future studies. Analyses of *CASC15* in adult B-ALL, AML, and correlations with clinicopathologic indicators will be important in defining a prognostic role for this lncRNA.

Here, we also identified a mouse transcript that shows sequence and functional conservation with human *CASC15*. Concordant with an overall role for *CASC15* as a tumor suppressor gene, our data also showed that overexpression of *CASC15* led to increased cell death in leukemia cell lines following prednisolone treatment. Additionally, primary hematopoietic cells that were transduced with mouse and human *CASC15* showed decreased colony formation and decreased levels of reconstitution in bone marrow transplantation assays. All of these findings suggest that *CASC15* expression may limit cell proliferation.

However, the high expression of *CASC15* in *RUNX1*-translocated cases is curious. Indeed, this might reflect a differential function in cell proliferation versus differentiation- with high *CASC15* causing a block in differentiation along with decreased proliferation. It is also possible that *CASC15* expression in leukemic cells simply reflects the stage of differentiation that they are derived from. In hematopoiesis, the expression profile of *CASC15* shows a bimodal distribution, with a peak in CLPs and a second peak in large pre-B-cells. Presumably, constitutive overexpression of *CASC15* prevents the drop seen at the pre-pro-B-cell stage and causes the observed myeloid bias and/or a block in B-cell development in the transplanted mice. It is possible that the cellular function of *CASC15* in modulating cell survival is also the basis of its function during development. Further studies, for example, in mice with deletion of *Bim* or overexpression of *Bcl2*, may help clarify the role of *CASC15* in differentiation versus cell survival.

Interestingly, our findings of reduced colony formation, reduced engraftment and myeloid bias have parallels with cellular and molecular phenotypes induced by expression of the *ETV6-RUNX1* fusion protein. For example, a series of studies have shown that the number of colonies produced by bone marrow transduced with *ETV6-RUNX1* was 10-fold reduced compared to control, showed myeloid bias, and showed an initial selective disadvantage in reconstitution of bone marrow [47–49]. Gene expression analysis has shown that ETV6-RUNX1 targets by ChIP-seq are enriched for inhibitors of proliferation pathways [50]. Hence, the mechanisms underlying transformation by ETV6-RUNX1 are complex, and require cooperating mutations and/or epigenetic events.

Our data also demonstrated knockdown of lncRNA utilizing the CRISPR-Cas9 system with a single guide RNA. Since we could not utilize traditional sgRNA targeting approaches for protein coding genes, which rely on generating frameshift mutations, we targeted transcription start, intron-exon junctions and poly A signal sites. Here, targeting three different intron-exon junctions in the *CASC15* transcript was sufficient to cause knockdown. Other manuscripts that successfully report lncRNA knockdown using CRISPR/Cas9 have utilized different strategies [51, 52]. By using this simple and very straightforward technique for designing small genomic RNAs (sgRNAs), this promising new technology can be used in generating lncRNA knockouts, and to study the function of lncRNAs in high-throughput screening approaches using a single sgRNA targeting individual lncRNAs. We also validated a more traditional strategy to create deletions of segments of DNA using CRISPR/Cas9 mediated targeting. By generating a single retroviral vector that can successfully carry two sgRNAs, we have developed a high-titer retroviral reagent for efficient knockdown of lncRNA expression. However, it should be noted that all of these methods create a mixture of heterozygous and homozygous knockouts cells, and hence the downstream confirmation of knockout remains important.

In line with a function in transcriptional regulation, genes that neighbor *CASC15* were downregulated by *CASC15* knockdown. However, the most consistent result to emerge from this study was the strong positive relationship between *CASC15* and *SOX4*. Found to be strongly correlated in primary human leukemic cells and in mouse cells, as well as in experimental datasets with knockdown and overexpression of *CASC15/Casc15*, our findings are consistent with studies that demonstrate that the expression of lncRNAs is tightly coupled to that of neighboring genes [2]. Moreover, *CASC15* knockdown led to altered expression of genes that were enriched for transcriptional targets of SOX4. These findings suggest that one of the major functions of *CASC15* may be to regulate the expression of *SOX4*. A potential mechanism is suggested by the fact that *CASC15* knockdown by any method led to alterations in the global transcriptome regulated by the transcription factor YY1. Our further functional analyses revealed that *CASC15* promotes YY1-mediated transcriptional activity at the SOX4 promoter. It will be of great interest to determine if *CASC15* globally modulates which promoters YY1 binds to, and whether this occurs through direct interaction or via modulation of general transcriptional complexes.

*SOX4* is thought to function as an oncogene in acute leukemia, particularly in the myeloid lineage [8, 53–55]. SOX4 expression is thought to support self-renewal of leukemic cells and to inhibit differentiation in C/EBP α-mutant AML [55], support PI3K/Akt signaling in *BCR-ABL*-driven B-ALL [53], and cooperate with *Pu.1* haploinsufficiency in murine leukemia [54]. However, in this study, *Casc15* increased Sox4 but caused decreased engraftment in bone marrow transplantation experiments. This may also be in line with patient-based studies that have shown that elevated expression of *SOX4* leads to better survival, decreased disease progression, and reduced tumor cell invasiveness in several different cancers [56–58]. Hence, both *CASC15*-mediated regulation and *SOX4* function may be dependent on the transcriptional context of the leukemic cell, and this has not been previously assessed in B-ALL with *RUNX1* or *MLL* translocations. Further work to characterize the normal function of *CASC15* in hematopoietic development using germline murine knockout models or via CRISPR/Cas9 deletion in bone marrow cells will help illuminate its role in hematopoiesis.

## Conclusion

Our work demonstrates cellular roles for a lincRNA identified in B-ALL. Additionally, we demonstrate a novel method of lincRNA knockdown by targeting splice junctions with the CRISPR/Cas9 system. At the molecular level, it appears that *CASC15* works by regulating expression of *SOX4*, likely by modulating the activity of transcription factors such as YY1. Our work opens the door to more extensive studies of lincRNAs in B-ALL and hematopoiesis. By better understanding lincRNA mediated actions in malignant gene expression programs, we will be better able to design prognostic indicators and therapeutic strategies targeting or harnessing lincRNAs.

## Additional files

Additional file 1: Table S1. Primers, siRNAs, guideRNAs and RACE sequences. (PDF 154 kb)

Additional file 2: Table S2. Antibodies used for FACS analyses. (PDF 105 kb)

Additional file 3: Figure S1. (A) *CASC15* expression is higher in RUNX1 translocated patients respect to other B-ALL subtypes with aberrancies related to chromosome 21. Probe 229280_s_at, HGU133 plus 2 Affymetrix (DS-ALL, Down Syndrome) (1- way ANOVA, *p* < 0.01). (B) *CASC15* expression is higher in RUNX1 translocated patients respect to other AML subtypes. Probe TC06000136.hg.1 HTA 2.0 Affymetrix. (1- way ANOVA, *p* < 0.01). Comparisons were made using a two-tailed T-test, statistically significant differences are denoted as follows: ** *P* < 0.01. Source of data for (A) and (B): two datasets deposited in NCBI’s Gene Expression Omnibus database (GEO) (*N* = 102 ALL, *N* = 85 AML; GSE17459 and GSE75461) [25, 26]. (C) Diagram showing 5′ and 3′ RACE product aligned with Ref sequence obtained from the UCSC genome browser. 5′ RACE primers are shown in blue. Unannotated exons are shown in yellow. (D-E) Gel showing 5 and 3′ RACE products. (F) Kaplan Meier survival analysis for two patient groups (high and low *CASC15* expressers) shows that low *CASC15* expression shows a trend towards worse overall survival (Log Rank Test, *P*-value, n.s. *p* = 0.18). The two groups were dichotomized based on two step cluster analysis using SPSS software. (G) Schematic showing the exon-intron structure of the two isoforms of *CASC15* (H) Schematic showing the exon-intron structure of the mouse *Casc15*. (I-K) RT-qPCR data showing expression of *CASC15* in cytoplasm and nuclear fractionations of from REH (I), RS4;11 (J) and 697 cell lines (K). Abbreviations: WCL (whole cell lysate), C (cytoplasmic fraction), and N (nuclear fraction). GAPDH and CELF4 were used as positive controls for cytoplasmic and nuclear-localized mRNAs, respectively [5]. 1 and 2 are biological replicates. (PDF 671 kb)

Additional file 4: Figure S2. (A) MTS assay showing no significant difference in cell proliferation in *CASC15(S)* over expressing NALM6 cells. B) PI staining of *CASC15(S)* over expressing NALM6 cells, showing no difference in the stages of cell cycle. C) FACS analysis of peripheral bleeds from the mice 4–20 weeks after bone marrow transplantation showing GFP positive cells as a percentage in the control and *Casc15* overexpression mice. Initial GFP positivity in the engrafted bone marrow was similar in both groups. (D) Complete blood counts (CBC) of control and *Casc15* overexpression mice at the week of 20 from the time of retro orbital injections. E) FACS analysis of Hardy fractions showing overall decreased B-cell fractions in *Casc15* overexpression mice at 27 weeks after transplantation. (F-G) FACS analysis of LIN- and LSK+ cells from the control and *Casc15* over expression mice showing no difference in those two populations. (H) Methylcellulose Colony Formation assay showing reduced number of colonies in BM cells with enforced expression of human *CASC15*. (I) FACS analysis of percentage of GFP+, B220+ and CD11b + cells in the spleen at the week of 16 after transplantation. Black circles, control mice; brown squares, *Casc15*-expressing mice. Data are represented as individual data points and a mean (bar). Statistical comparisons were completed using a two-tailed T-test; *p* < 0.05 (*); *p* < 0.01 (**); *p* ≤ 0.0005 (***). (PDF 89 kb)

Additional file 5: Figure S3. (A). RT-qPCR showing the expression of *PRL* in RS4;11 cell line and *LOC79217* in REH and RS4;11 cells. Statistical comparisons were completed using a two-tailed T-test; *p* < 0.05 (*); *p* < 0.01 (**); *p* ≤ 0.0005 (***). (B) Correlation between *SOX4* and *CASC15* expression in ETV6-RUNX1-translocated primary B-ALL samples (left panel), B-ALL cell lines (middle panel) and AML samples (right panel). (C) Correlation between *SOX4* and *CASC15* expression in publically available datasets (Cancer cell line encyclopedia) [29] in AML cell lines (top left), B-ALL cell lines (top right), DLBCL (bottom left) and other non-hematopoietic cell lines (bottom right). High degrees of correlation are seen in AML and B-ALL cell lines. (D) MTS assay showing no significant difference cell proliferation upon *CASC15* knockdown by siRNA 1-2in RS4;11 cell line. (E) Strategy to knockout *CASC15* using CRISPR/Cas9-mediated gene editing. Target sites that were utilized are denoted, superimposed on the exon-intron structure of *CASC15*. (F)RT-qPCR to measure *CASC15* expression following CRISPR/Cas9-mediated gene editing of *CASC15* in RS4;11 cells. (G-J)T7 Endonuclease assay showing the presence of heteroduplex DNA generated by CRISPR-Cas9-mediated cleavage at the transcription start at exon 1 (C1) (G), splice junction at exon 9 (C9) (H), exon 11 (C11) (I) and poly A signal site (C12) (J). T7 enzyme cleavage is detected by the presence of multiple bands in the C1, C9, C11 and C12 integrated cells compared to the vector. (PDF 742 kb)

Additional file 6: Figure S4. (A, B) Schematics (A) and FACS plots (B) showing the sorting strategy for B-cell progenitor fractions as per the method of Hardy et al. [59, 60]. (PDF 250 kb)

Additional file 7: Figure S5. (A) Heat map comparison of gene expression in REH cells transduced with LentiCRISPR versus those transduced sgRNA against exons 1, 9 of *CASC15* (See Fig. 3). Columns represent technical replicates utilized with Affymetrix U133 human chip. (B) Disease association analysis was carried out using Webgestalt, http://www.webgestalt.org. Shown are the numbers of disease-associated genes in each disease that showed a statistically significant association with which the differentially expressed gene set in *CASC15* KO REH cells. (C) GSEA was performed on the differentially expressed gene set in *CASC15* KO REH cells, showing a significant association with the transcriptome regulated by *SOX4*, as also noted in the RS4;11 KD cells (Fig. 4). Left panel: Enrichment score (ES) -0.38 and FDRq = 0.017. Right panel: ES: −0.4526 and FDRq = 1. (D) Transcriptional activity of the *SOX4* promoter with increasing levels of *CAC15* transfected into HEK-293 T cells, as measured by dual luciferase assay. (E) Results of RIP assay: Western blot characterization of immunoprecipitate from YY1 pull-down (top panel) and RIP enrichment, determined as RNA associated to YY1, relative to IgG control (bottom panel). (PDF 546 kb)

## Abbreviations

AML: Acute myeloid leukemia
B-ALL: B-acute lymphoblastic leukemia
CCLE: Cancer cell encyclopedia
CLP: Common lymphoid progenitor
CMV: Cytomegalovirus promoter
GEO: Gene Expression Omnibus database
HSC: Hematopoietic stem cell
LMPP: Lymphoid primed multipotent progenitor
LSK: lineage- Sca1^+^ c-Kit^+^
MSCV: Murine Stem Cell Virus
ORF: Open reading frame
pre-B: precursor B
pro-B: Progenitor B
RACE: Rapid amplification of cDNA ends
RBC: Red blood cell
RIP: RNA Immuno Precipitation
SOX4: SRY-Box 4
YY1: Yin and Yang-1
