# Supplementary material for: The lncRNA CASC15 regulates SOX4 expression in RUNX1-rearranged acute leukemia
Source: Mol Cancer. 2017 Jul 19;16:126. doi: 10.1186/s12943-017-0692-x (PMC5517805; doi:10.1186/s12943-017-0692-x)
Supplement: Supplementary file 1 — Primers, siRNAs, guideRNAs and RACE sequences. (PDF 154 kb) [file 12943_2017_692_MOESM1_ESM.pdf]

RT-qPCR primers		
CASC15	FOW1	5’ CGCCGGGGTATCTCCCTCTCG 3’
	REV1	5’ CATTTCCCCCGCTGCAGTCCA 3’
	FOW2	5’ TGGAGTCCAAGTGTGACTGCCAAG 3’
	REV2	5’ TCCTCTGGGTTTTCCATGCGTGT 3’
SOX4	FOW	5’ AGCGACAAGATCCCTTTCATTC 3’
	REV	5’ CGTTGCCGGACTTCACCTT 3’
PRL1	FOW	5’ GGGGTTCATTACCAAGGCCA 3’
	REV	5’ TGGATAGGATAGCCTCCGGG 3’
MYC	FOW	5’ CCACAGCAAACCTCCTCACAG 3’
	REV	5’ GCAGGATAGTCCTTCCGAGTG 3’
ACTIN	FOW	5' CATGTACGTTGCTATCCAGGC 3'
	REV	5' CTCCTTAATGTCACGCACGAT 3'
RAPGEF4	FOW	5’ CACCTCTCATTGAACCTCACG 3’
	REV	5’ GTCCGGGAGTGAACACATGG 3’
JUN	FOW	5' TCCAAGTGCCGAAAAAGGAAG 3'
	REV	5' CGAGTTCTGAGCTTTCAAGGT 3'
LOC729177	FOW	5’ AGATCACAGTGGACAGCCACTCA 3’
	REV	5’ CAAATATTGGCTGCCTTAAGCTCTC 3’
L32 (mouse)	FOW	5' AAGCGAAACTGGCGGAAAC 3'
	REV	5' TAACCGATGTTGGGCATCAG 3'
Casc15	FOW	5’ TGCAAGTTAGCACTGGACAA 3’
	REV	5’ AGAATTCTCGGGCATCACACT 3’

**mmu-miR-155 formatted siRNA oligos**	
CASC15-siRNA1	5’<br>GAAGGCTGTATGCTGAAAGTTGACCCTCAAGTCTGAGTTTTGGCCACTGACTGACTCAGACTT<br>GGGTCAACTTTCAGGACACAAGGCCTG 3’
CASC15-siRNA2	5’<br>GAAGGCTGTATGCTGTTGGCAGTCACACTTGGACTCGTTTTGGCCACTGACTGACGAGTCCAA<br>GTGACTGCCAACAGGACACAAGGCCTG 3’
CASC15-siRNA3	5’<br>GAAGGCTGTATGCTGAAGAAATCAAGCCTGCCCATAGTTTTGGCCACTGACTGACTATGGGCA<br>CTTGATTTCTTCAGGACACAAGGCCTG 3’
CASC15_C1-1	CACCGTCCCGGAGCAGACAAAGAGG
CASC15_C1-2	AAACCCTCTTTGTCTGCTCCGGGAC
CASC15_C9-1	CACCGAGGAAGTTGAAGATTTGTCC
CASC15_C9-2	AAACGGACAAATCTTCAACTTCCTC
CASC15_C11-1	CACCGGCCAATACCCACCAGAAGCA
CASC15_C11-2	AAACTGCTTCTGGTGGGTATTGGCC
CASC15_C12-1	CACCGTATTATTGGTATAATCATCA
CASC15_C12-2	AAACTGATGATTATACCAATAATAC
5’ RACE Sequence<br>CASC15	Product 1:<br>NNNNNNNNNTCTCNGGGTTNNNTGCGTGTGGCGTGCTTCGGGCAGGGCTACATGC<br>TTGGGCTTTTTGGCTTTGCTGGATTCCTCTTGGCAGTCACACTTGGACTCCACAGCT<br>CCATTAGCCACTTCCTTCTTCTGTCTGTTTGTTTTGAGGACATCTGGTGAAACAGAC<br>AATGGAGAACAGCCATCCATGGGTCGGGGGGAATTGAGAAGGATGTTCAGTAGT<br>AACCCAGGGATGACTTGGTGACGGAAAAGAAGGCACACATTTTTAAATCTGCATA<br>GGACATCAAATTAGATGTGGTTGAGCAGTAAATGCAGATCCTTCAGCTGGTAAAG<br>TTGACCCTCAAGTCTGAGCTGAAGATTTTCTCAGAAACTTTCAAATCAAGATCTAT<br>ATCTCCACAGATAACAATAATGGAGTGACTCCACCANTAGATTTTACTCGTCTCAG<br>GTAATTCTGCTGGAAAGGGAAGGAAGTAAATCTTGGANTCCTGCACTTCCATCCG<br>TTAATCATATACATGCACGCCCTCAAAAGCANTAAATCACTGTCTTTCACTGTATT<br>GCCATGATTCACACCTGAAGAGCACTCATCAGTCATTCTGACTGTCTTGTGATAAT<br>GCTTCAGCAAACCCACCAGTGAAATGAGCTTGTTGCCAATTCCTGGAAATCTTCCC<br>CATCCAGAAAGTCTCCTACCGGAACATGCTGGGTGTTAACACATCTGTAGTCACCT<br>GAGGGTACTTCTTTAAGGTGGAGAATTCCCAGCGATCAAATTGTAATCCATATAN<br>AAAANGTATCATCAAAAATTCCAATGATTTAACTTTTAAGCAACTCCAGATGAAT<br>CCAGGAATGATTTGACCTTGTATTGGTACTTCCATATTGTTGGCTGATGGAAAGAT<br>CCAGTAAGAACAACGCAGAATGAAGGAGGTCAGTGCTAGGAAGTACTCCAGCAA<br>ATTCATGATGCTTTCTAGATGCTGATAATAGTAATTACACCTTCTTCCCCACTTCGT<br>CGCACTTTTAAGGGACATTTCCCCCGCTGCAGTCCACGAGGCGTTCGGACACTTTT<br>TCCCGGCCTGACAG

	Product 2:<br>NNNNNNNNNCTCTGGGTTNNNTGCGTGTGGCGTGCTTCGGGCAGGGCTACATGCT<br>TGGGCTTTTGGCTTTGCTGGATTCCTCTTGGCAGTCACACTTGGACTCCACAGCTC<br>CTTGACTCTGTTTATCAAGGGAGGAATCCCGACAGCATGGAGCCCCCAGGCCTCC<br>CCTCCTCCATCAGCACAGCATGCCCGCAGTCACAAGCTGCCTGCGAGTTTGGTAA<br>GACTCTGGCTCCAATCCCTTGGCCTCATCTGACCCGTCTTTCTGCCGTATCACTTAT<br>CTCCTGGAAAGGAACATGTTTCCAAGCTTCTTCCACATCAGCAGTAGGAAAGGAA<br>TTTGCAGTCTTTGAGTATTAAGTGACCATTAGCCACTTCCTTCTTCTGTCTGTTTGT<br>TTTGAGGACATCTGGTGAAACAGACAATGGAGAACAGCCATCCATGGGTCGGGGG<br>GAATTGAGAAGGATGTTCAGTAGTAACCCAGGGATGACTTGGTGACGGAAAAGA<br>AGGCACACATTTTAAATCTGCAGAGGACATCAAATTAGATGTGGTTGAGCAGTAA<br>ATGCAGATCCTTCAGCTGGTAAAGTTGACCCTCAAGTCTGAGCTGAAGATTTTCTC<br>AGAAACTTTCAAATCAAGATCTAGATCTCCACAGATAAGAATAATGGAGTGACTC<br>CACCAGTAGATTTTACCTGTTGAGTTAGGAGGGTAGGAATGCAGGAAGGACAGAG<br>GCAGGAAACAACAGTTTGGAGAACTTCTGTCCCANGCTTGAAACCCTCTTTATAA<br>CATTCCTCTCCGTCACCTTTCACATCTTCACAGTCACTAAGTCCTGACTTTTCTGTT<br>TCATCAAAGCCAGCAAACGCAGTGTTCGGATCCGCGA
3’ RACE Sequence CASC15	Product1:<br>NNNNNNNNNNNNNNNNNNNNNNNNNNNNNNNGCCTNNGAGTTCTCATTTNTGTTNNCCTCTGG<br>AAACCACATGTGTACCTTGTTTGCAACAGTATGGGTTCGGANGCNNAAAGAATTTTCCTTGTCTTGGA<br>TGAGACTTTTGACTTNTTNTTTTGGGTTAANTTCTNNAGAGTCTCCTTGCTTCTGATTCTACATCTCCAT<br>CCATGGGCCACTGTTTCAACAACCTCANCCAGTGCAACACAACCTCATCCAAGAAGAGTATGCAGAGA<br>AAGGACTCCCCTACCTGCCACAAAACTGTTGTCTGAAAACTGTCTCATATTGTCACAAGTTGTCATTCA<br>TTGTGAATTACACCTGTTTAACATGTAATCTGCAACATGCTTCACTGTCTAATTTTCCAGAGCCCCTCAT<br>ATAAGGAACTGTATTATTGGTATAATCATCATGGTGAAGAAGTTGGTNTTTCCAGAACCCCTCAT<br><br>Product2:<br>NNNNNNNNNNNNNNNNNNNTNNTNNTGAGAAGTTGGTANNNGGGGGAGAGANGA<br>CAGAAACAGAGAGTAAGTCAGAGCTGGCTGCCTGACAGATAAAAAGGAAATGAC<br>CAAAAAAAAAAAAACCTATNNNGNNTNNNATTAATTCGGATCCNGGNN

Table S1: Primers, siRNAs, guideRNAs and RACE sequences
