# Supplementary material for: The lncRNA CASC15 regulates SOX4 expression in RUNX1-rearranged acute leukemia
Source: Mol Cancer. 2017 Jul 19;16:126. doi: 10.1186/s12943-017-0692-x (PMC5517805; doi:10.1186/s12943-017-0692-x)
Supplement: Supplementary file 2 — Antibodies used for FACS analyses. (PDF 105 kb) [file 12943_2017_692_MOESM2_ESM.pdf]

Fraction	Subset	Marker Profile
A	Pre-Pro-B	$B220^+$, $CD43^+$, $CD24^-$, $Ly51^-$
B	Pro-B	$B220^+$, $CD43^+$, $CD24^+$, $Ly51^-$
C	Pro-B/ Large pre-B	$B220^+$, $CD43^+$, $CD24^+$, $Ly51^+$
D	Small Pre-B	$B220^+$, $CD43^-$, IgM^-
E	Immature B	$B220^+$, $CD43^-$, IgM^+

Full definition	Abbreviation	Marker Profile
Hematopoietic Stem Cell	HSC	Lin^-, c-Kit ^{high}, Sca-1^{high}, $CD150^+$
Lymphoid-primed Multipotent Progenitors	LMPP	Lin^-, c-Kit ^{high}, Sca-1^{high}, $Flt3^+$, IL-7$R\alpha^-$
Common Lymphoid Progenitor	CLP	Lin^-, c-Kit ^{low}, Sca-1^{low}, $Flt3^+$, IL-7$R\alpha^+$
		* CD3e, CD8a, CD4, Gr-1, IgM, B220, CD11b, Ter119, NK-1.1, TCRγδ, TCR-β

* Lin markers

Supplementary Table 2: Antibodies used for FACS analyses.
