# Supplementary figures and images for: The lncRNA CASC15 regulates SOX4 expression in RUNX1-rearranged acute leukemia
Source: Mol Cancer. 2017 Jul 19;16:126. doi: 10.1186/s12943-017-0692-x (PMC5517805; doi:10.1186/s12943-017-0692-x)

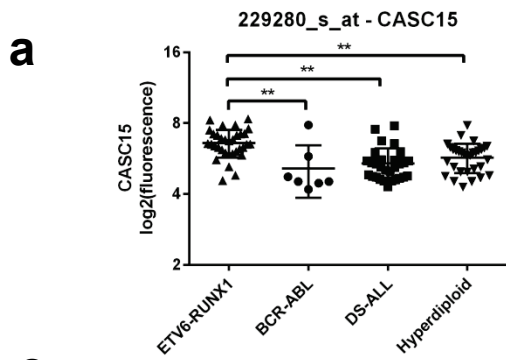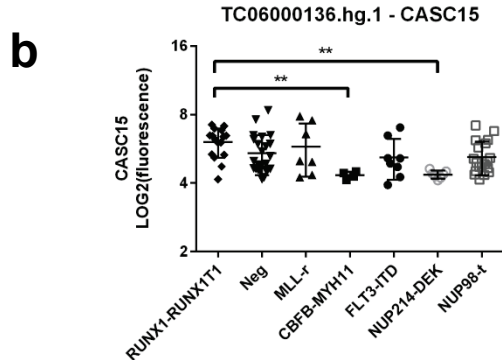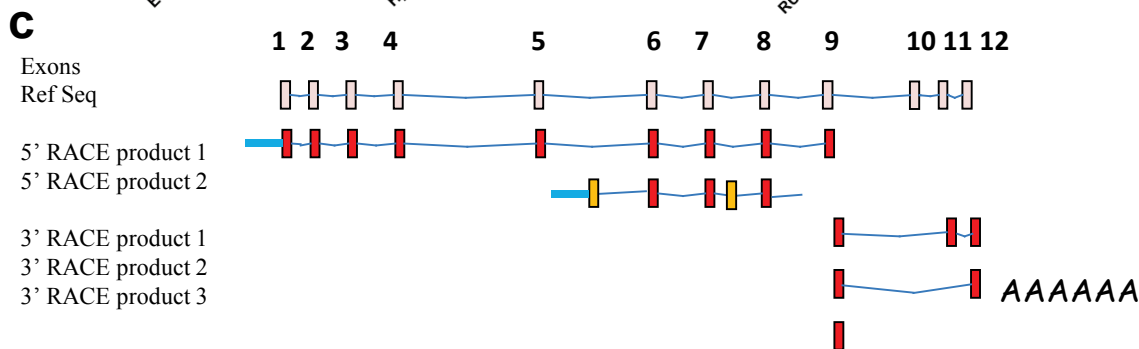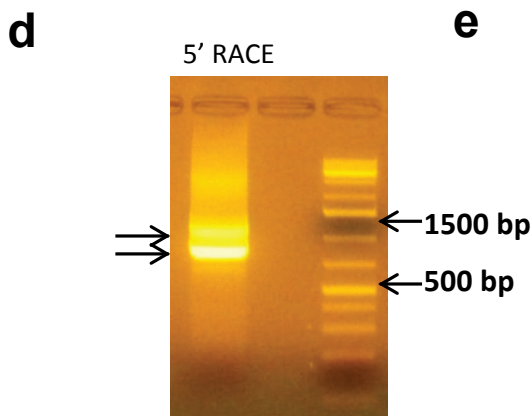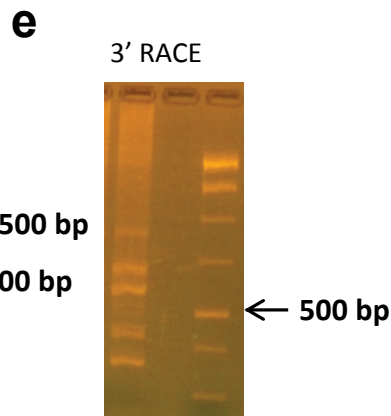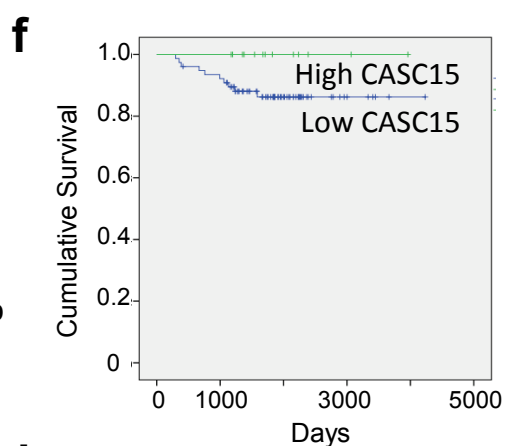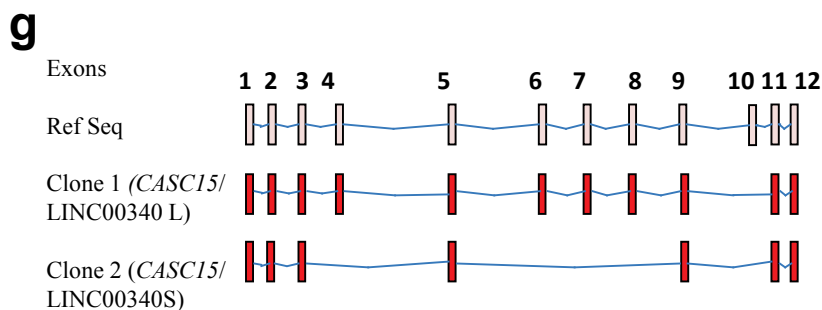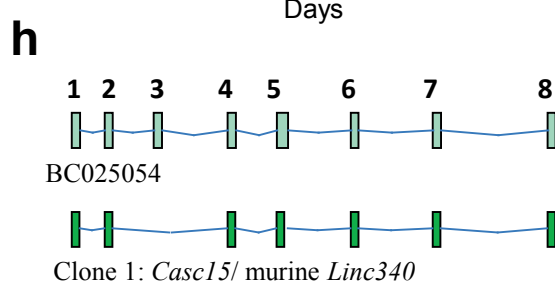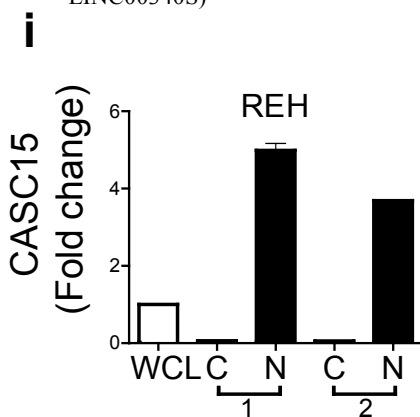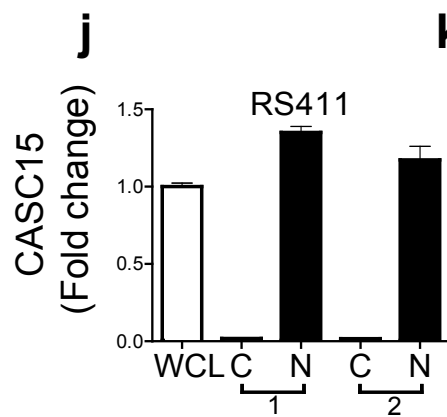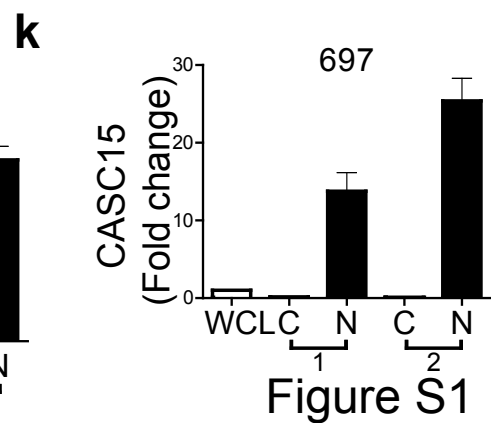

Supplement: Supplementary file 3 — (A) CASC15 expression is higher in RUNX1 translocated patients respect to other B-ALL subtypes with aberrancies related to chromosome 21. Probe 229280_s_at, HGU133 plus 2 Affymetrix (DS-ALL, Down Syndrome) (1- way ANOVA, p < 0.01). (B) CASC15 expression is higher in RUNX1 translocated patients respect to other AML subtypes. Probe TC06000136.hg.1 HTA 2.0 Affymetrix. (1- way ANOVA, p < 0.01). Comparisons were made using a two-tailed T-test, statistically significant differences are denoted as follows: ** P < 0.01. Source of data for (A) and (B): two datasets deposited in NCBI’s Gene Expression Omnibus database (GEO) (N = 102 ALL, N = 85 AML; GSE17459 and GSE75461) [25, 26]. (C) Diagram showing 5′ and 3′ RACE product aligned with Ref sequence obtained from the UCSC genome browser. 5′ RACE primers are shown in blue. Unannotated exons are shown in yellow. (D-E) Gel showing 5 and 3′ RACE products. (F) Kaplan Meier survival analysis for two patient groups (high and low CASC15 expressers) shows that low CASC15 expression shows a trend towards worse overall survival (Log Rank Test, P-value, n.s. p = 0.18). The two groups were dichotomized based on two step cluster analysis using SPSS software. (G) Schematic showing the exon-intron structure of the two isoforms of CASC15 (H) Schematic showing the exon-intron structure of the mouse Casc15. (I-K) RT-qPCR data showing expression of CASC15 in cytoplasm and nuclear fractionations of from REH (I), RS4;11 (J) and 697 cell lines (K). Abbreviations: WCL (whole cell lysate), C (cytoplasmic fraction), and N (nuclear fraction). GAPDH and CELF4 were used as positive controls for cytoplasmic and nuclear-localized mRNAs, respectively [5]. 1 and 2 are biological replicates. (PDF 671 kb) [file 12943_2017_692_MOESM3_ESM.pdf]

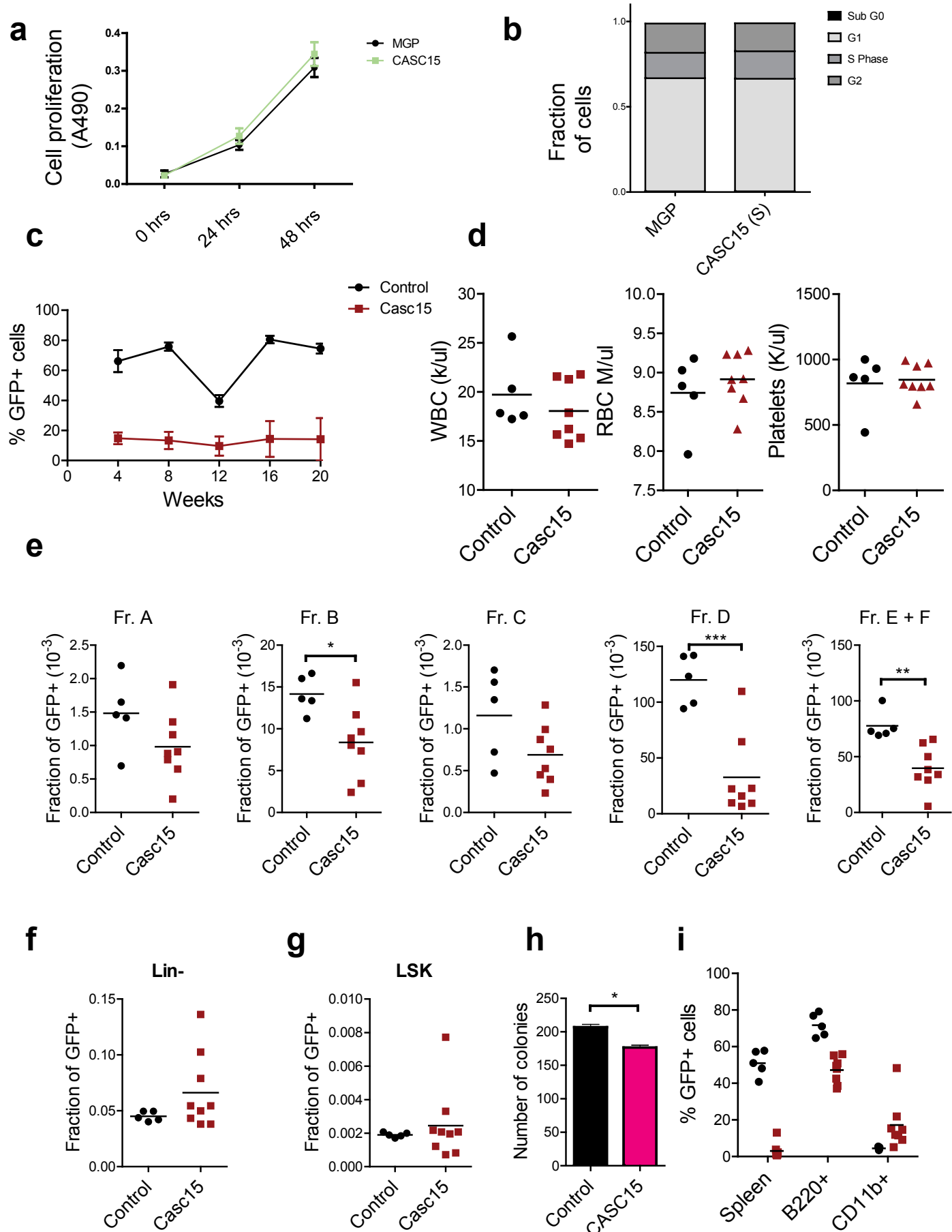

Figure S2

Supplement: Supplementary file 4 — (A) MTS assay showing no significant difference in cell proliferation in CASC15(S) over expressing NALM6 cells. B) PI staining of CASC15(S) over expressing NALM6 cells, showing no difference in the stages of cell cycle. C) FACS analysis of peripheral bleeds from the mice 4–20 weeks after bone marrow transplantation showing GFP positive cells as a percentage in the control and Casc15 overexpression mice. Initial GFP positivity in the engrafted bone marrow was similar in both groups. (D) Complete blood counts (CBC) of control and Casc15 overexpression mice at the week of 20 from the time of retro orbital injections. E) FACS analysis of Hardy fractions showing overall decreased B-cell fractions in Casc15 overexpression mice at 27 weeks after transplantation. (F-G) FACS analysis of LIN- and LSK+ cells from the control and Casc15 over expression mice showing no difference in those two populations. (H) Methylcellulose Colony Formation assay showing reduced number of colonies in BM cells with enforced expression of human CASC15. (I) FACS analysis of percentage of GFP+, B220+ and CD11b + cells in the spleen at the week of 16 after transplantation. Black circles, control mice; brown squares, Casc15-expressing mice. Data are represented as individual data points and a mean (bar). Statistical comparisons were completed using a two-tailed T-test; p < 0.05 (*); p < 0.01 (**); p ≤ 0.0005 (***). (PDF 89 kb) [file 12943_2017_692_MOESM4_ESM.pdf]

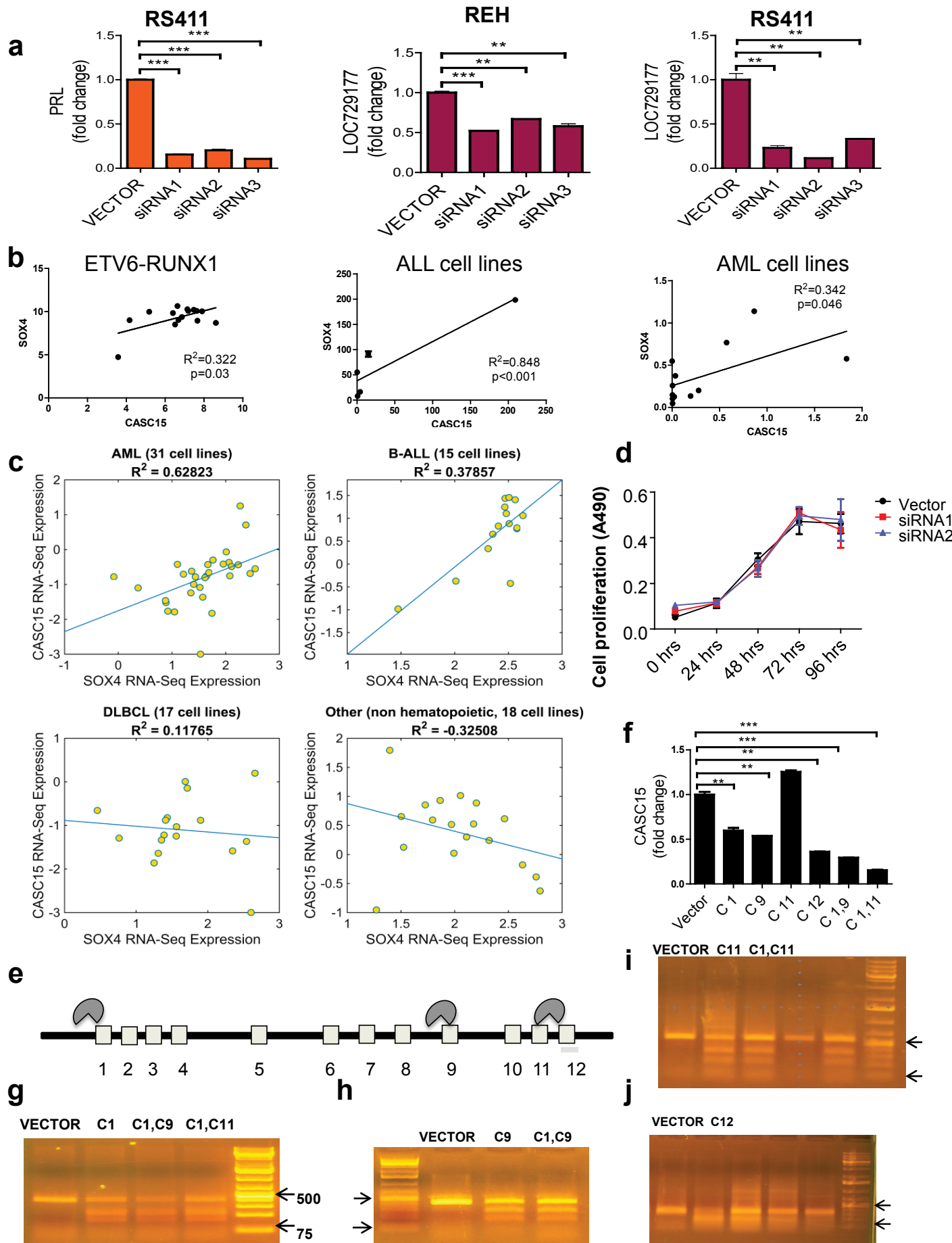

Figure S3

Supplement: Supplementary file 5 — (A). RT-qPCR showing the expression of PRL in RS4;11 cell line and LOC79217 in REH and RS4;11 cells. Statistical comparisons were completed using a two-tailed T-test; p < 0.05 (*); p < 0.01 (**); p ≤ 0.0005 (***). (B) Correlation between SOX4 and CASC15 expression in ETV6-RUNX1-translocated primary B-ALL samples (left panel), B-ALL cell lines (middle panel) and AML samples (right panel). (C) Correlation between SOX4 and CASC15 expression in publically available datasets (Cancer cell line encyclopedia) [29] in AML cell lines (top left), B-ALL cell lines (top right), DLBCL (bottom left) and other non-hematopoietic cell lines (bottom right). High degrees of correlation are seen in AML and B-ALL cell lines. (D) MTS assay showing no significant difference cell proliferation upon CASC15 knockdown by siRNA 1-2in RS4;11 cell line. (E) Strategy to knockout CASC15 using CRISPR/Cas9-mediated gene editing. Target sites that were utilized are denoted, superimposed on the exon-intron structure of CASC15. (F)RT-qPCR to measure CASC15 expression following CRISPR/Cas9-mediated gene editing of CASC15 in RS4;11 cells. (G-J)T7 Endonuclease assay showing the presence of heteroduplex DNA generated by CRISPR-Cas9-mediated cleavage at the transcription start at exon 1 (C1) (G), splice junction at exon 9 (C9) (H), exon 11 (C11) (I) and poly A signal site (C12) (J). T7 enzyme cleavage is detected by the presence of multiple bands in the C1, C9, C11 and C12 integrated cells compared to the vector. (PDF 742 kb) [file 12943_2017_692_MOESM5_ESM.pdf]

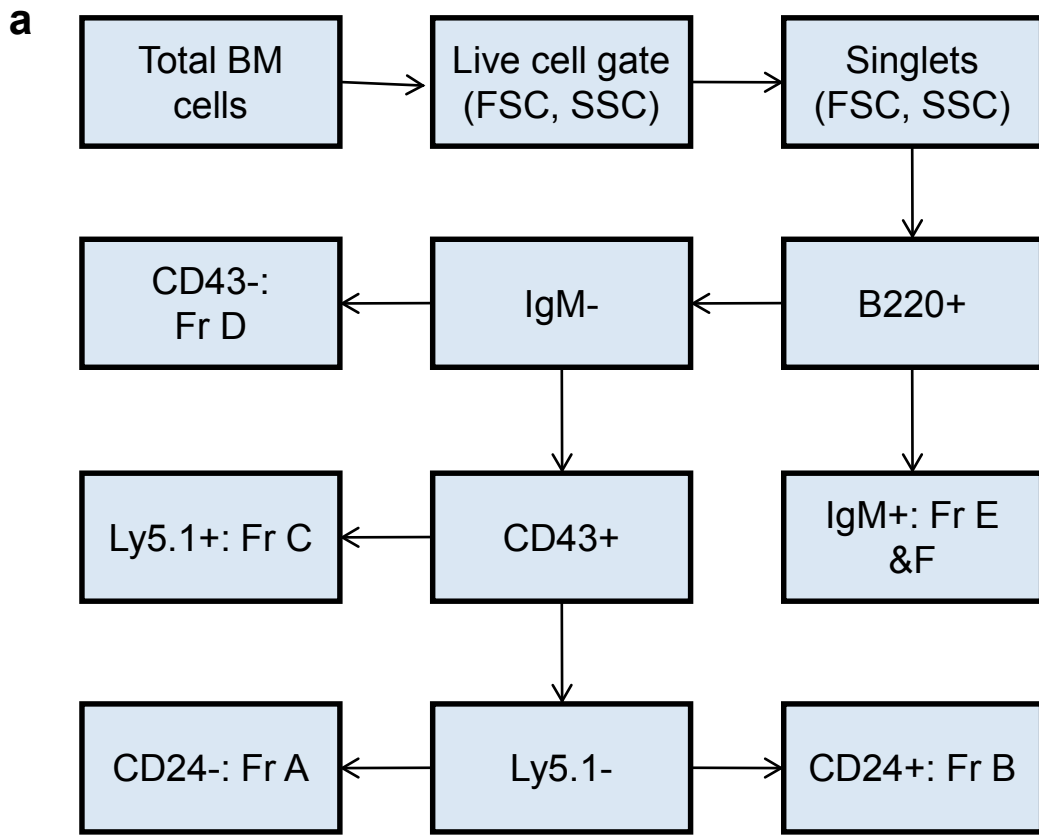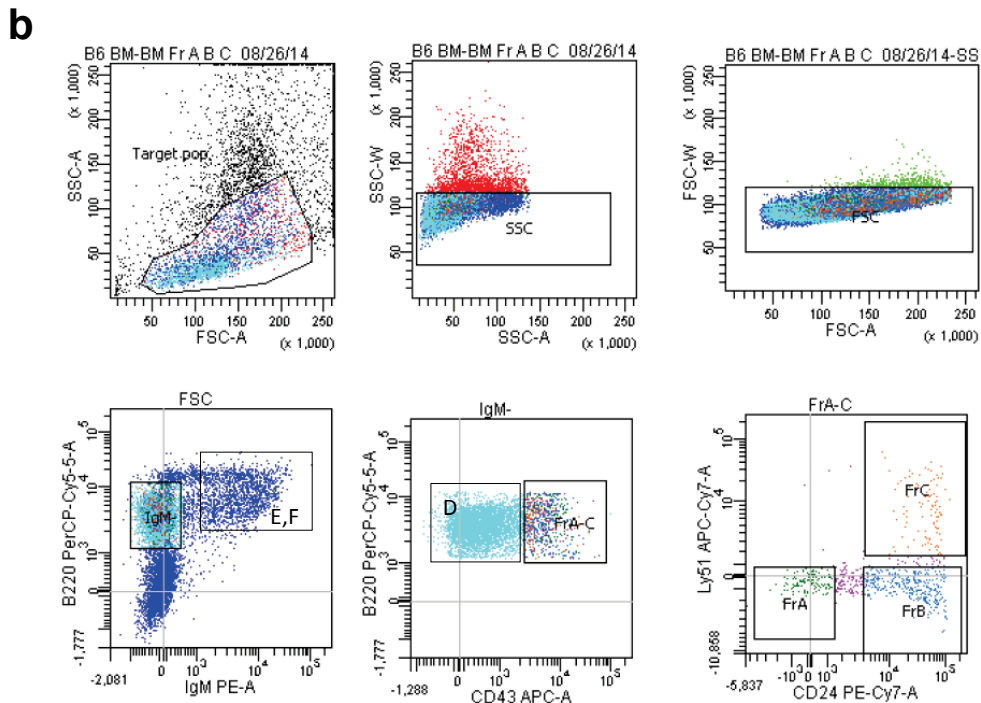

Figure S4

Supplement: Supplementary file 6 — (A, B) Schematics (A) and FACS plots (B) showing the sorting strategy for B-cell progenitor fractions as per the method of Hardy et al. [59, 60]. (PDF 250 kb) [file 12943_2017_692_MOESM6_ESM.pdf]

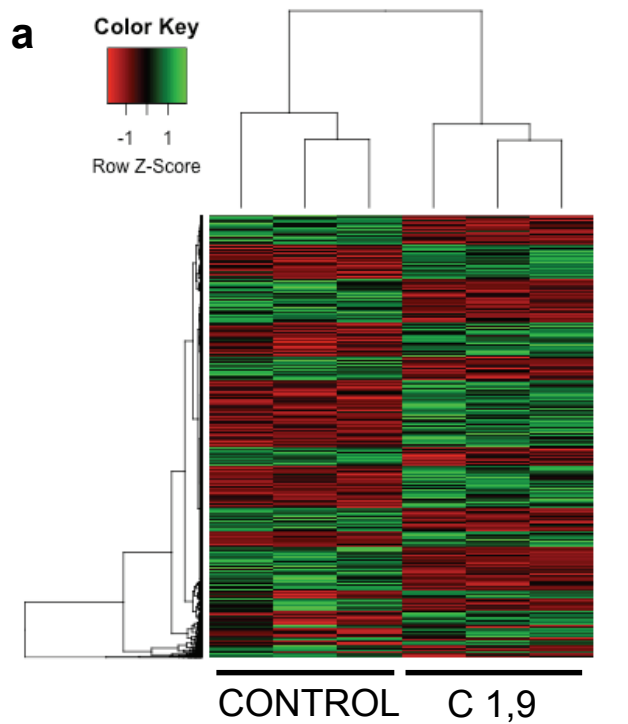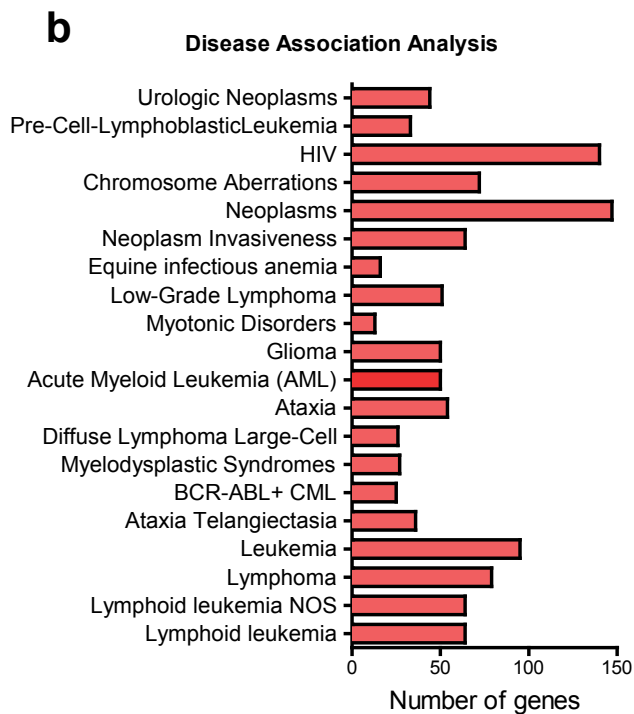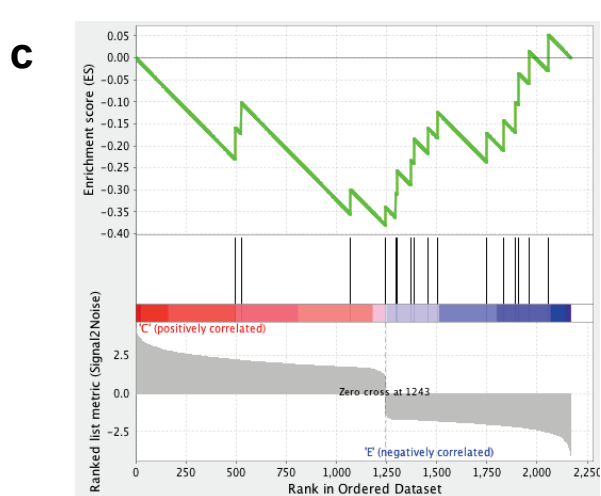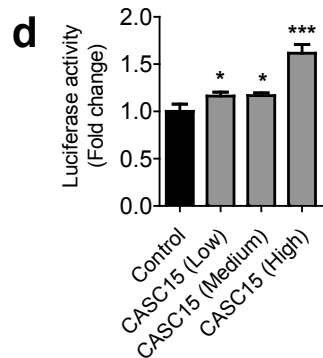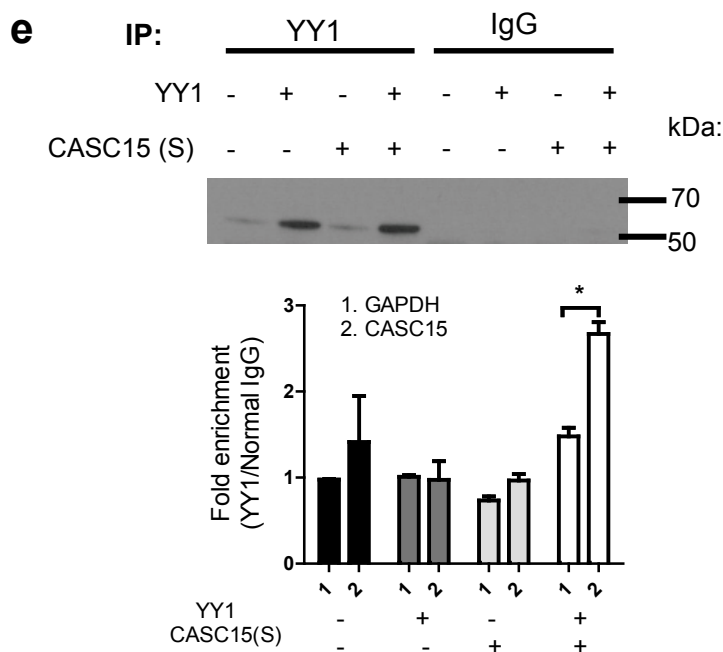

**Figure S5**

Supplement: Supplementary file 7 — (A) Heat map comparison of gene expression in REH cells transduced with LentiCRISPR versus those transduced sgRNA against exons 1, 9 of CASC15 (See Fig. 3). Columns represent technical replicates utilized with Affymetrix U133 human chip. (B) Disease association analysis was carried out using Webgestalt, http://www.webgestalt.org. Shown are the numbers of disease-associated genes in each disease that showed a statistically significant association with which the differentially expressed gene set in CASC15 KO REH cells. (C) GSEA was performed on the differentially expressed gene set in CASC15 KO REH cells, showing a significant association with the transcriptome regulated by SOX4, as also noted in the RS4;11 KD cells (Fig. 4). Left panel: Enrichment score (ES) -0.38 and FDRq = 0.017. Right panel: ES: −0.4526 and FDRq = 1. (D) Transcriptional activity of the SOX4 promoter with increasing levels of CAC15 transfected into HEK-293 T cells, as measured by dual luciferase assay. (E) Results of RIP assay: Western blot characterization of immunoprecipitate from YY1 pull-down (top panel) and RIP enrichment, determined as RNA associated to YY1, relative to IgG control (bottom panel). (PDF 546 kb) [file 12943_2017_692_MOESM7_ESM.pdf]
